# Urinary phytate concentration and risk of fracture determined by the FRAX index in a group of postmenopausal women

**DOI:** 10.3906/sag-1806-117

**Published:** 2019-04-18

**Authors:** ANGEL A. LOPEZ-GONZALEZ, FELIX GRASES, BARTOLOME MARÍ, MATIAS TOMÁS-SALVÁ, ADRIAN RODRIGUEZ

**Affiliations:** 1 Occupational Health Investigation Group, University Institute of Health Sciences Research (IUNICS), University of the Balearic Islands, Palma Spain; 2 Laboratory of Renal Lithiasis Research, University Institute of Health Sciences Research (IUNICS), University of the Balearic Islands, Palma Spain

**Keywords:** Phytate, postmenopausal women, fracture risk, FRAX index, hip fracture

## Abstract

**Background/aim:**

This study was designed to evaluate the relationship between urinary phytate concentration and risk of fracture at 10 years, determined by using the FRAX model, in women who had undergone menopause within 5 years of the time of enrollment.

**Materials and methods:**

Of the 212 postmenopausal women evaluated, 69 were excluded because they had urinary phytate concentrations between 0.51 and 0.99 mg/L. Of the remaining 143 women, 91 had low (≤0.50 mg/L) and 52 had high (≥1.0 mg/L) urinary phytate concentrations. The 10-year risk of fracture was calculated by using the FRAX model.

**Results:**

The risks of major osteoporotic fracture and hip fracture were higher in women with low urinary phytate levels (P < 0.001 in both cases). Evaluation of the risk of hip fracture in women with and without risk factors for osteoporosis (e.g., tobacco, alcohol, and drug consumption) and according to urinary phytate concentrations indicated that, among women with no risk factors, those with low and high urinary phytate levels had a range of risks of 0%–0.6% and 0%–0.3%, respectively (P = 0.098). Moreover, among women with at least one risk factor, those with low and high urinary phytate had a range of risks of 0.1%–0.8% and 0.1%–0.4%, respectively (P = 0.002). Similar results were observed when the risks of major osteoporotic fracture were analyzed.

**Conclusion:**

These results indicate the relationship of phytate with the risks of major osteoporotic fracture and hip fracture, with these differences being more marked in women with risk factors for osteoporosis. From this study follows the importance of the consumption of phytate-rich products (nuts, legumes, whole cereals) to protect against the risk of fracture in 10 years, mainly in women with risk factors for osteoporosis.

## 1. Introduction

Osteoporosis has been defined as a skeletal disease characterized by reduced bone strength, thereby predisposing individuals to an increased risk of fracture (1). Bone strength can be determined by measuring the bone density (in grams of mineral per unit area or volume) and the properties of bone and tissue, including its macro- and microarchitecture. However, reduced bone strength is not the only factor predictive of the risk of fracture, with several factors not associated with bone being predictive of the risk of fracture.

Based on observational studies, various clinical practice guidelines have concluded that the most important risk factors in the development of fractures include personal history of a fragility fracture, defined as a spontaneous fracture during adult life or a fracture caused by trauma that would not have occurred in a healthy individual; age ≥65 years; history of fragility fracture in at least one first-grade relative; low body mass index (BMI; <20 kg/m2); and glucocorticoid treatment (2–4). High risk factors include falling in the previous year, hypoparathyroidism, eating disorders, and chronic malnutrition and malabsorption. Moderate risk factors include female sex; early menopause, at age 40–45 years; consumption of >3 units of alcohol/day; being a current smoker; and having insulin-dependent diabetes, rheumatoid arthritis, or hyperthyroidism. 

Risk of fracture is associated with bone mineral density (BMD). Dual-energy X-ray absorptiometry (DXA) has been found to be optimal in measuring BMD of the spine and hips to predict the risk of fracture (4,5). The combination of clinical risk factors for fragility fracture with measurement of BMD by central DXA has been shown to be the most effective predictor of the risk of fracture (2). 

The FRAX model is an assessment tool for fracture risk in men and women aged 40–90 years (6). Model algorithms using computer programs that calculate the 10-year probabilities of overall brittle fractures and hip fractures have been developed for 12 countries: Austria, China, France, Germany, Italy, Japan, Spain, Sweden, Switzerland, Turkey, the United Kingdom, and the United States (with specific models for persons of Caucasian, African American, Latino, and Asian ethnicities). These models have been validated in cohort studies and found to be accurate in predicting the 10-year risks of overall and hip fractures.

Myo-inositol hexaphosphate, also called phytic acid and phytate, is a molecule abundant in vegetable seeds, like legumes, nuts, and whole cereals (7). Phytate constitutes 1.5%–6.4% of the dry weight of grains and is mostly bound to calcium and magnesium ions (7). Phytate is also found in mammalian tissues and organs at levels dependent on dietary intake (8), with correlations between concentrations in plasma and urine (9). Phytate, which acts as an antioxidant, has been found to affect human health (7). For example, phytate has beneficial effects on renal lithiasis (10,11), sialolithiasis (12), tartar (13), and cardiovascular calcifications (14–16). Moreover, phytate apparently has protective effects against osteoporosis (17-20). Therefore, this study evaluated the relationship of phytate with fracture risk as assessed by the FRAX index.

## 2. Materials and methods

### 2.1. Participants

This descriptive cross-sectional pilot study included 212 postmenopausal women from Majorca (Balearic Islands). Subjects were evaluated with in-depth clinical interviews, including whether or not they had entered menopause; in the case of doubt, hormone concentrations were measured. Personal and clinical data were collected by GESMA’s Occupational Health Service. The study protocol was approved by the Balearic Research Ethics Board (Protocol #IB 1027/08 PI). Considering our previous experience related to urinary phytate levels, the participants in the present study were divided into three groups: one with low urinary phytate levels (≤0.5 mg/L); one with medium urinary phytate levels (0.51–0.99 mg/L), which were excluded; and one with high urinary phytate levels (≥0.99 mg/L). Criteria for inclusion and exclusion are shown in Table 1.

**Table 1:** Criteria for subjects’ acceptance into the study.

Inclusion criteria	Exclusion criteria
Female sex	>5 years since menopause
Occurrence of menopause	Surgical menopause
Agreement to participate in the study and provision of informed consent	Treatment with bisphosphonates or other drugs used to treat osteoporosis
	Urinary phytate concentrations between 0.51 and 0.99 mg/L

### 2.2. Urine samples and phytate analysis

All women consumed an unrestricted diet during the sample collection period. A urine sample was collected from each volunteer. Two hours after the first urine of the morning, a fasting urine sample was collected, stored at 4 °C, and transported to the laboratory in a chilled container. Phytate concentrations were immediately analyzed as described (21). The method used to determine phytate is nonspecific, so in addition to inositol hexaphosphate (Insp6) it can also include the determination of other lower inositol phosphates (InsP5, InsP4, etc.) (7).

### 2.3. Determination of bone mineral density

BMD of the femoral neck was determined by DXA (Norland Excell bone densitometer, MEC Osteoporosis Bone Densitometry, Minster, OH, USA) and is reported as g/cm2. All densitometry measurements were performed by a single technician to avoid interobserver bias. 

### 2.4. Evaluation of fracture risk using FRAX

Demographic and clinical data collected included patient age; parental history of osteoporotic hip fracture; personal history of fragility fracture; current consumption of tobacco; consumption of 3 or more units/day of alcohol, with each unit defined as 8–10 g of alcohol; rheumatoid arthritis; and use of oral corticosteroids for more than 3 months (≥5 mg/day of prednisolone or equivalent doses of other glucocorticoids). 

Factors associated with secondary osteoporosis were also recorded, including histories of type 1 diabetes, imperfect osteogenesis in adults, untreated chronic hyperthyroidism, hypogonadism, early menopause (before 45 years of age), chronic malnutrition, malabsorption, and chronic liver disease. Weight and height were determined by approved and calibrated scales and BMI was calculated. The FRAX index for each subject was calculated using a computer tool available online for the Spanish population (22). The 10-year probabilities of hip fracture and of major osteoporotic fractures, defined as the sum of clinical vertebral, forearm, hip, and proximal humerus fractures, were calculated.

### 2.5. Statistics

Normality of the data was assessed by the Kolmogorov–Smirnov test and plotting of histograms and Q-Q graphs. As the majority of the data were not normally distributed, continuous variables were expressed as medians and interquartile ranges, or ranges (minimum and maximum values). Between groups, the differences were assessed by the Mann–Whitney U test. Risks and qualitative variables were expressed as percentages. Qualitative variables were compared between groups using the chi-square test. All statistical analyses were performed using SPSS 22 for Windows. 

## 3. Results

Of the 212 women evaluated, 69 had urinary phytate concentrations between 0.51 and 0.99 mg/L and were excluded. Of the remaining 143 subjects, 91 had low (≤0.50 mg/L) and 52 had high (≥1.0 mg/L) urinary phytate concentrations. The demographic and clinical characteristics of these groups are shown in Table 2. Of the factors analyzed, only BMD of the femoral neck differed significantly in the two groups.

**Table 2:** Demographic and clinical characteristics of postmenopausal women with low and high urinary phytate concentrations.

	Low urinary phytate (≤0.5 mg/L)a	High urinary phytate (≥1.0 mg/L)a	P-valueb
Age (years)	52 (4)	51 (5)	0.389
Weight (kg)	65.0 (9)	64.5 (9)	0.807
Height (cm)	160 (8)	161 (8)	0.987
BMI (kg/m2)	24.7 (4.2)	24.6 (4.2)	0.906
Years since menopause	3 (1)	4 (2)	0.252
BMD femoral neck (g/cm2)	0.72 (0.14)	0.84 (0.14)	<0.001
Fracture history (%)	19.8	17.3	0.716
Tobacco consumption (%)	30.8	34.6	0.636
Glucocorticoids (%)	5.5	5.8	0.945
Alcohol (%)	12.1	11.5	0.922
Secondary causes of osteoporosis (%)	8.8	7.7	0.820

aMedian (interquartile range) or percentage. bComparison of low and high urinary phytate groups. Mann–Whitney U test or chi-square test.

**Figure 1:**
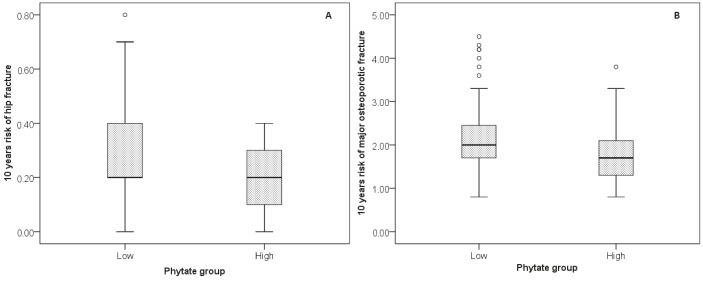
Box-plots for (A) 10-year risk of hip fracture and (B) 10-year risk of major osteoporotic fracture, relative to urinary phytate concentration.

The 10-year risk of hip fracture was significantly higher in subjects with low than with high urinary phytate levels (ranges: 0%–0.8% and 0%–0.4%, P < 0.001; Figure 1A).

Similarly, the 10-year risk of major osteoporotic fractures (proximal humerus, clinical vertebral forearm, hip) were significantly higher in subjects with low than with high urinary phytate levels (ranges: 0.8%–4.5% and 0.8%–3.8%, P < 0.001; Figure 1B).

**Figure 2:**
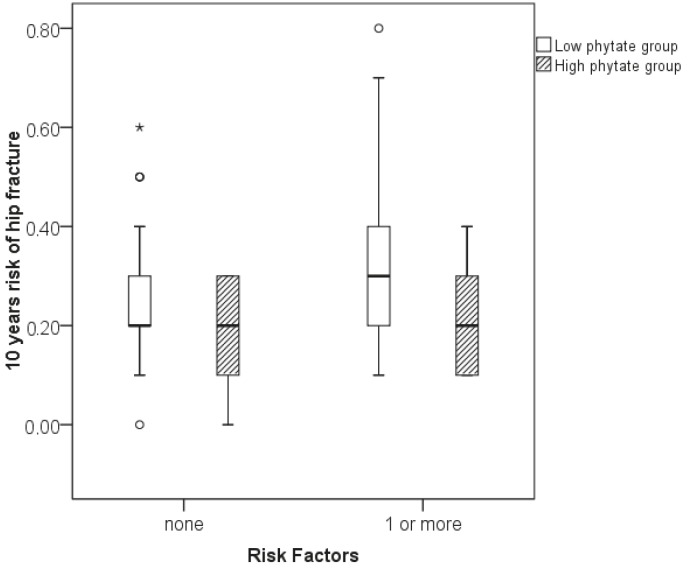
Box-plots for 10-year risk of hip fracture relative to urinary phytate concentrations and presence or absence of risk factors for osteoporosis.

Evaluation of the risk of hip fracture in women with and without risk factors for osteoporosis (e.g., tobacco, alcohol, and drug consumption) and according to urinary phytate concentrations showed that, among women with no risk factors, those with low and high urinary phytate levels had a range of risks of 0%–0.6% and 0%–0.3%, respectively (P = 0.098; Figure 2). Moreover, among women with at least one risk factor, those with low and high urinary phytate had a range of risks of 0.1%–0.8% and 0.1%–0.4%, respectively (P = 0.002; Figure 2).

**Figure 3:**
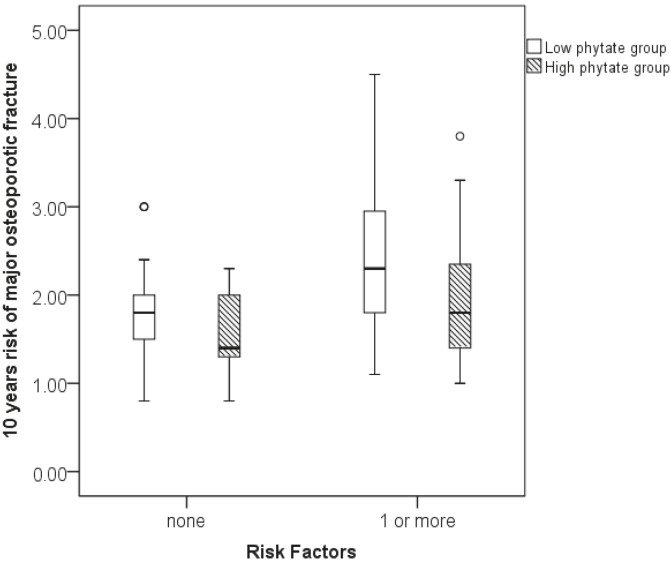
Box-plots for 10-year risk of major osteoporotic fracture relative to urinary phytate concentration and presence or absence of risk factors for osteoporosis.

In the same way, evaluation of the risk of major osteoporotic fractures in women with and without risk factors for osteoporosis and according to urinary phytate concentration showed that, among women with no risk factors, those with low and high urinary phytate levels had a range of risks of 0.8%–3% and 0.8%–2.3% respectively (P = 0.041; Figure 3). Moreover, among women with at least one risk factor, those with low and high urinary phytate had a range of risks of 1.1%–4.5% and 1.0%–3.8%, respectively (P = 0.005; Figure 3).

## 4. Discussion

Phytate consumption by postmenopausal women living on Mallorca reduced their risks of hip and major osteoporotic fractures, as calculated by the FRAX model. To the best of our knowledge, the current assessment of the relationship between urinary phytate and the risks of fracture in postmenopausal women is the first study to examine this link in any population, including other groups such as nonmenopausal women and men. 

Previous studies by our group showed a direct relationship between the consumption of phytate, as determined by food surveys, and increased BMD in the calcaneus, lumbar spine, and femoral neck in the general population (17) and in postmenopausal women (20). Similar results were observed when phytate consumption was determined by measuring urinary phytate concentrations (18) using an unspecific method that evaluates the set of inositol phosphates, as well as in ovariectomized rats (19). Another study found that bone loss in postmenopausal women was lower in the group of women that consumed greater amounts of phytate (20). The classification of participants according to low urinary phytate (<0.5 mg/L), medium urinary phytate (0.51–0.99 mg/L), and high urinary phytate (<0.99 mg/L) is a consequence of the results obtained in the previous studies that have been cited. The results of the present study therefore provide further evidence that consumption of phytate increases BMD, slows bone loss, and reduces the risk of fractures at various locations in postmenopausal women. Interestingly, these positive effects of phytate consumption were greater in women with risk factors for osteoporosis.

The effects of phytate are similar to those of some bisphosphonates, which are chemically similar to phytate. Phosphate and phosphonate groups have high affinities for calcium crystal surfaces, thereby promoting self-adsorption and avoiding redissolution (23). In this aspect it is important to note that the analytical method that has been used to analyze phytate in urine is a nonspecific method to evaluate inositol hexaphosphate (InsP6), and also evaluates the presence of other inositol phosphates (InsP5, InsP4, etc.), which, being polyphosphates, can also play an important role in avoiding the redissolution of hydroxyapatite, in a manner analogous to bisphosphonates. These substances also reduce the activities of osteoclasts, thereby protecting against bone reabsorption (24,25).

The main limitation of the present study was its inclusion of only women with a maximum of 5 years since menopause. The beneficial effects of phytate consumption on bone mass and fracture risk should also be assessed in other subjects, including women >5 years past menopause, premenopausal women, and men.

In conclusion, phytate consumption by postmenopausal women reduced their risks of hip and major osteoporotic fractures, as calculated by the FRAX model. The positive effects of phytate consumption were greater in women with than without risk factors for osteoporosis. The main limitation of the present study was the inclusion only of women with a maximum of 5 years since menopause.
